# ClearSight™ finger cuff versus invasive arterial pressure measurement in patients with body mass index above 45 kg/m^2^

**DOI:** 10.1186/s12871-021-01374-x

**Published:** 2021-05-18

**Authors:** Victoria Eley, Rebecca Christensen, Louis Guy, Kerstin Wyssusek, Anita Pelecanos, Benjamin Dodd, Michael Stowasser, Andre van Zundert

**Affiliations:** 1 Department of Anaesthesia and Perioperative Medicine, The Royal Brisbane and Women’s Hospital, Butterfield St, Herston, Queensland 4006 Australia; 2 Faculty of Medicine, The University of Queensland, St Lucia, Queensland 4067 Australia; 3 Statistics Unit, Queensland Institute of Medical Research Berghofer, Herston, Brisbane, 4006 Australia; 4 Department of Surgery, The Royal Brisbane and Women’s Hospital, Butterfield St, Herston, Queensland 4006 Australia; 5 Hypertension Unit, Princess Alexandra Hospital, Woolloongabba, Brisbane, 4102 Australia

**Keywords:** ClearSight™, Invasive blood pressure, Non-invasive blood pressure, Obesity, Vascular unloading

## Abstract

**Background:**

Measuring blood pressure in patients with obesity is challenging. The ClearSight™ finger cuff (FC) uses the vascular unloading technique to provide continuous non-invasive blood pressure measurements. We aimed to test the agreement of the FC with invasive radial arterial monitoring (INV) in patients with obesity.

**Methods:**

Participants had a body mass index (BMI) ≥45 kg/m^2^ and underwent laparoscopic bariatric surgery. FC and INV measurements were obtained simultaneously every 5 min on each patient, following induction of anesthesia. Agreement over time was assessed using modified Bland-Altman plots and error grid analysis permitted clinical interpretation of the results. Four-quadrant plots allowed assessment of concordance in blood pressure changes.

**Results:**

The 30 participants had a median (IQR) BMI of 50.2 kg/m^2^ (IQR 48.3–55.3). The observed bias (SD, 95% limits of agreement) for systolic blood pressure (SBP) was 14.3 mmHg (14.1, -13.4 – 42.0), 5.2 mmHg (10.9, -16.0 – 26.5) for mean arterial pressure (MAP) and 2.6 mmHg (10.8, -18.6 – 23.8) for diastolic blood pressure (DBP). Error grid analysis showed that the proportion of readings in risk zones A-E were 90.8, 6.5, 2.7, 0 and 0% for SBP and 91.4, 4.3, 4.3, 0 and 0% for MAP, respectively. Discordance occurred in ≤8% of pairs for consecutive change in SBP, MAP and DBP.

**Conclusions:**

The vascular unloading technique was not adequately in agreement with radial arterial monitoring. Evaluation in a larger sample is required before recommending this technique for intraoperative monitoring of patients with BMI ≥45 kg/m^2^.

## Background

Accurate non-invasive blood pressure (NIBP) measurement is an important component of perioperative care. This measurement is known to be difficult patients with Class III obesity [1]. Traditional rectangular NIBP cuffs may be inadequate due to the large mid-arm circumference [2] or the aberrant shape of the arm [3]. As the arm becomes more “cone-shaped”, NIBP measurement error increases [4].

The ClearSight™ EV1000 Clinical Platform (Edwards Lifesciences Corp, Irvine, CA) [5] provides continuous NIBP measurement using the vascular unloading technique [6] via a finger cuff (FC). The device has largely been evaluated in non-obese surgical patients [7–9]. When assessed in 50 patients with body mass index (BMI) < 40 kg/m^2^ undergoing cardiothoracic surgery, Martina and colleagues found that the FC met Association for the Advancement of Medical Instrumentation (AAMI) criteria for validation [8]. However in 112 non-obese patients undergoing non-cardiac surgery, the FC failed to meeting AAMI criteria for validation against invasive measurements [9].

The evaluation of ClearSight™ in patients with obesity was limited until recently, when Rogge et al. evaluated ClearSight™ [10] and Schumann et al. evaluated ccNexfin™ (BMEYE B.V., The Netherlands; predecessor of ClearSight™) [11] in patients with obesity undergoing bariatric surgery. Rogge et al. suggested good accuracy and precision for mean arterial pressure and diastolic blood pressure [10]. Schumann et al. demonstrated differences (mean, SD) of -1 mmHg (± 11) for mean arterial pressure, -7 mmHg (± 14) for systolic blood pressure, and 0 mmHg (± 11) for diastolic blood pressure between the finger cuff and invasive measurements. Both authors suggested minimal clinical risk from use of the finger cuff in patients with obesity [10, 11].

Devices utilising FCs rather than oscillometric NIBP cuffs have potential benefits by avoiding the variation in arm shape that is observed in patients with obesity [3]. Accurate blood pressure measurement in patients with obesity pre-operatively or post-operatively can be difficult or impossible due to arm morphology [1]. Invasive monitoring is reasonably considered inappropriate in these contexts, due to risk to patients and institutional policies. FCs could potentially fulfil this unmet need.

We aimed to compare the accuracy of the continuous NIBP measurements obtained via a FC with the gold standard, invasive radial arterial blood pressure monitoring (INV), in patients with BMI ≥45 kg/m^2^. The primary aim was to assess the agreement of the FC with INV measurements over time, including systolic blood pressure (SBP), mean arterial pressure (MAP) and diastolic blood pressure (DBP). An error grid analysis was undertaken to permit clinical interpretation of our results. We performed a four-quadrant plot, reporting on concordance, to evaluate the ability of the FC to track changes in blood pressure. In addition, we sought to describe the arm morphology of our participants in terms of mid-arm circumference and conicity index.

## Methods

This prospective methods comparison study was approved by the Human Research Ethics Committee of The Royal Brisbane and Women’s Hospital, Brisbane, Australia, (HREC/17/QRBW/165, 17/05/2017; Protocol Version 2.0) and participants provided written informed consent. This manuscript adheres to the STROBE guideline. We included participants if they were scheduled for elective laparoscopic bariatric surgery, were aged ≥18 years and had a BMI ≥45 kg/m^2^. A BMI > 40 kg/m^2^ is considered Class III obesity. We excluded participants if they had a known cardiac arrhythmia, were American Society of Anesthesiologists physical status [12] ≥ 4, had Raynaud’s phenomenon or other contraindications to radial arterial catheterisation. We documented participant characteristics, including age, sex, ethnicity, type of surgery, height, weight, BMI and the presence of diagnosed hypertension.

Measurements were obtained from both upper limbs according to standard anthropometric procedures [13], by two trained research personnel using a standard medical measuring tape. The arm length (L) was measured with the participant standing with the elbow flexed and held by the side, on the posterior aspect of the arm. The length was measured from the uppermost edge of the posterior border of the spine extending from the acromion process, to the tip of the olecranon process. With the arm hanging loosely by the side, the mid-arm circumference was measured at the mid-point of the arm length. In this position, the proximal arm circumference (C1, a non-standard measurement) was obtained at the axilla and the distal arm circumference (C2, a non-standard measurement) was obtained just above the elbow crease. These non-standard measurements were used to obtain arm diameters (D1 and D2), to be used in the calculation of the conicity index [4]. Conicity index = 100 x (D1 - D2)/L [4]. The conicity index increases as the arm becomes more cone-shaped [4].

Prior to induction, we inserted a radial arterial catheter. We placed the FC on the same side as the arterial line, on the middle phalanx of the middle finger using the appropriately-sized cuff and zeroed using the heart reference sensor. The cuff was selected using the finger-sizing guide provided by the manufacturer [5]. Where the size of the finger was larger than indicated on the sizing guide, this was noted and the largest finger cuff used. FC measurements were obtained from a free-standing ClearSight™ module, software suite version Piek 3. The invasive transducer (Edwards Lifesciences TruWave™, Edwards Lifesciences Corp, Irvine, CA, USA) was zeroed according to manufacturer’s instructions [14] and the vent port placed at the level of the right atrium. INV measurements were obtained from a D19KT™ monitor with a E-PSMP Carescape Module™ (GE Healthcare, Chicago, IL, USA). The arterial catheter was flushed and the waveform observed to allow detection and resolution of under- or over-damping.

General anesthesia was induced and maintained according to the preference of the specialist anaesthetist. No blood pressure targets were specified. It is our institutional practice to use vasopressor (metaraminol or phenylephrine) to maintain blood pressure during laparoscopic bariatric surgery and we documented the details of vasopressor use. Following standard positioning of the patient (one pillow under the head, table in the reverse Trendelenburg position) the first blood pressure measurements were obtained (T0). These included the SBP, DBP and MAP from the FC and INV measurement devices. Pressures were recorded digitally at 5 min intervals for each patient up to 1 hour, or until completion of anesthesia and stored for analysis.

The primary outcome was the agreement of the FC with INV measurements (MAP, SBP and DBP) at 5 min intervals during general anesthesia. Secondary outcomes included: a clinical interpretation of the data using an error grid analysis and a concordance analysis using four-quadrant plots to evaluate the ability of the FC to track arterial pressure changes.

### Statistical methods

Participant characteristics were summarised using descriptive statistics; mean and standard deviation (SD) for continuous normally distributed variables, median and interquartile range (IQR) for non-normally distributed variables and frequency and percent for categorical variables.

The data structure over time consisted of paired INV and FC measurements of MAP, SBP and DBP taken at 5-min intervals for each patient from commencement of anesthesia for 60 min or completion of general anesthesia, whichever occurred first. Agreement between INV and FC for MAP, SBP and DBP over the time points was assessed using the modified true value varies method of the Bland-Altman analysis for multiple observations per participant [15, 16]. The standard deviation of the bias and 95% limits of agreement were reported and the bias interpreted in the context of pre-specified acceptable limits of ±5 mmHg. This was selected to be consistent with recommendations of the AAMI for validating blood pressure monitoring devices [17]. We calculated the 95% confidence intervals for the limits of agreement according to the method of variance estimates recovery (MOVER) method, which takes into account the repeated measurements taken [18]. All assumptions of the Bland-Altman analysis were satisfied.

An error grid analysis was undertaken, based on the work of Saugel et al. [19]. This method allows the interpretation of the clinical relevance of the difference between the readings obtained from the FC and INV. Based on the expert opinion of specialists experienced in perioperative or critical care, Saugel et al. described five levels of clinical risk. The levels are based on whether or not the difference between the readings would trigger a therapeutic intervention and the potential consequences of that intervention; for example giving a treatment for hypotension, when the gold standard indicated that the blood pressure was normal or high. The risk levels are defined as [19]:

A. No risk (i.e. no difference in clinical action between the reference and test method)
B. Low risk (i.e. test method values that deviate from the reference but would probably lead to benign or no treatment)
C. Moderate risk (i.e. test method values that deviate from the reference and would eventually lead to unnecessary treatment with moderate non–life-threatening consequences for the patient)
D. Significant risk (i.e. test method values that deviate from the reference and would lead to unnecessary treatment with severe non–life-threatening consequences for the patient)
E. Dangerous risk (i.e. test method values that deviate from the reference and would lead to unnecessary treatment with life-threatening consequences for the patient)

We calculated the percentage of blood pressure readings corresponding to the risk levels A-E and the actual values were represented visually as five zones on the error grid. Clinically acceptable targets were considered to be less than 5% of readings in Zone B, 4% in Zone C and 2% in Zone D [19].

Changes in blood pressure between consecutive time points (5 min intervals) for FC and INV methods were plotted against each other in four-quadrant plots, reporting on concordance. An exclusion zone of +/− 5 mmHg was set, indicating only small changes in blood pressure with random noise. R (v3.6.0; R Core team, 2019, Vienna, Austria) was used for all analyses.

## Results

**Fig. 1:**
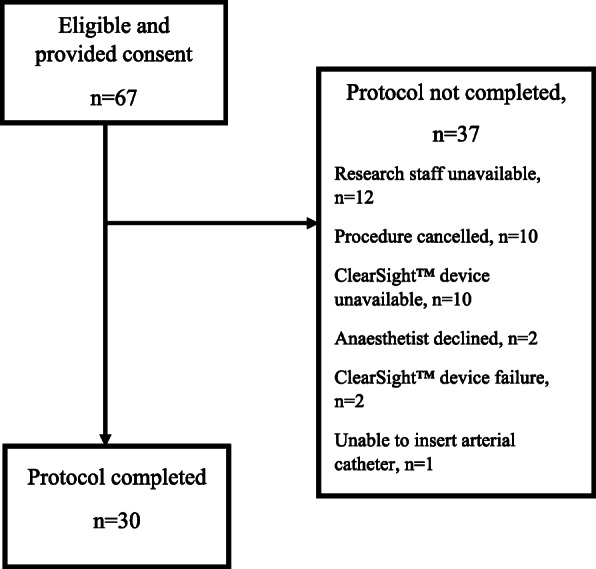
Recruitment flowchart

Thirty participants were eligible and completed the protocol between September 2018 and November 2019. Figure 1 shows reasons for failure to complete the protocol. The participants had a mean ± SD age of 45 ± 11.7 years (range 24 to 65), median BMI of 50.2 kg/m^2^ (IQR 48.3 to 55.3, range 45.1–69.2), 26 (87%) were female and 4 (13%) were male. Table 1 shows the demographic and arm morphology details of the participants. In 4 (13%) participants the circumference of the finger was larger than the manufacturer’s recommendation for the largest finger cuff. The INV measurement was obtained from the ipsilateral arm to the FC measurement in all cases. Vasopressor was used in 23 (77%) participants, with 13 (43%) administered metaraminol by infusion, 9 (30%) phenylephrine by infusion and 1 (3%) ephedrine by bolus.

**Table 1:** Characteristics of 30 patients presenting for elective bariatric surgery

Characteristic	Description
Weight, (kg) median (IQR)	146 (133 to 151)
BMI category (kg/m^2^) n (%)	
45.0–49.9	14 (47)
50.0–54.9	7 (23)
55.0–59.9	5 (17)
≥ 60.0	4 (13)
Diagnosis of hypertension n (%)	10 (33)
Ethnicity n (%)	
Caucasian	28 (93)
Aboriginal or Torres Strait Islander	2 (7)
Type of laparoscopic surgery n (%)	
Sleeve gastrectomy	10 (33)
Roux-en-y gastric bypass	10 (33)
Single anastomosis gastric bypass	5 (17)
Remove gastric band and convert to gastric bypass	4 (13)
Removal of gastric band	1 (3)
Right mid-arm circumference (cm), mean ± SD (range)	47.2 ± 6.3 (35–61)
Left mid-arm circumference (cm), mean ± SD (range)	46.4 ± 5.7 (38–58)
Right arm conicity index, mean ± SD (range)	14.9 ± 5.0 (4.9–24.3)
Left arm conicity index, mean ± SD (range)	15.3 ± 4.4 (8.5–22.7)
Vasopressor total dose	
Metaraminol (mg), median (IQR) (*n* = 13)	3.5 (2.0 to 6.2)
Phenylephrine (mcg), median (IQR) (*n* = 9)	955 (450 to 2210)
Ephedrine (mg), individual value presented	6

The total number of individual blood pressure measurements across MAP, SBP and DBP for both INV and FC was 2229. All patients had complete blood pressure measurements up to 15 min, after which the number of measurements per method ranged between 26 and 30, due to variations in the duration of surgery.

**Fig. 2:**
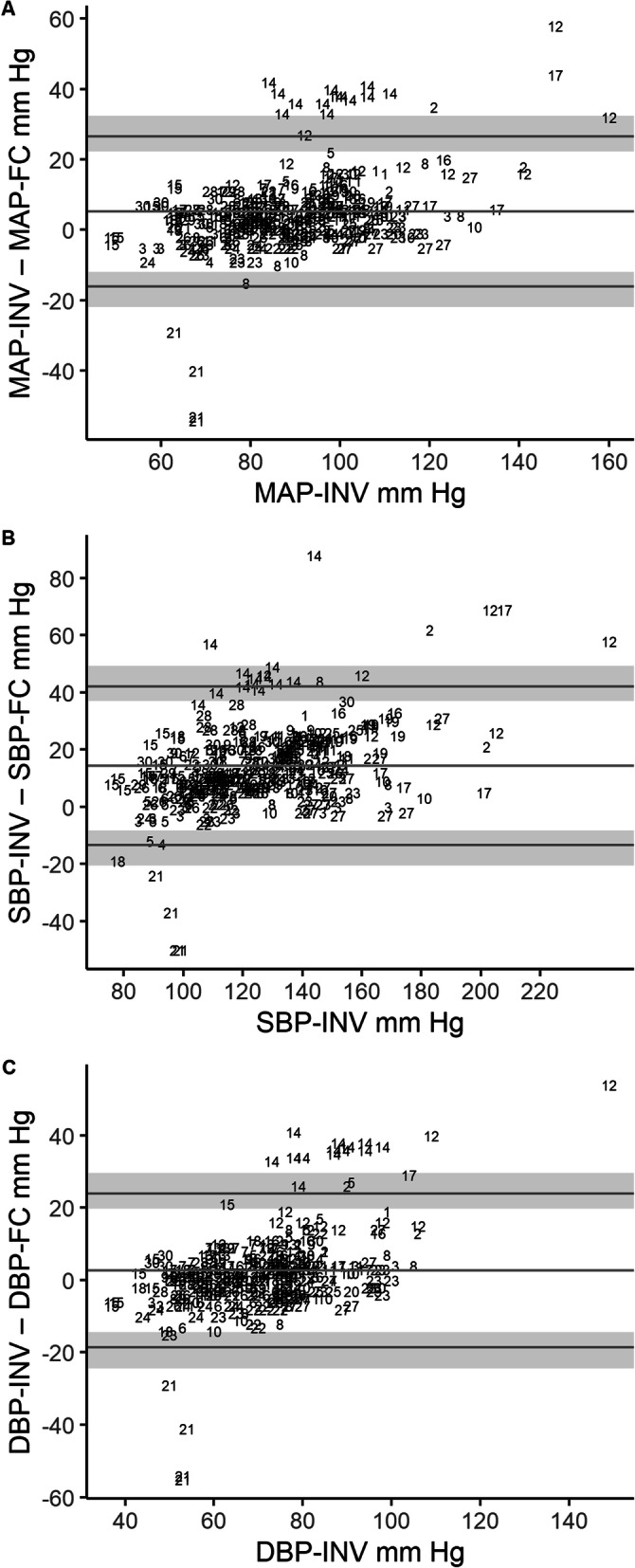
**a** Modified Bland-Altman plot for mean arterial pressure (MAP). The plot shows the agreement between measurements from invasive radial arterial monitoring (MAP-INV) and the finger cuff (MAP-FC). **b** Modified Bland-Altman plot for systolic blood pressure (SBP). The plot shows the agreement between measurements from invasive radial arterial monitoring (SBP-INV) and the finger cuff (SBP-FC). **c** Modified Bland-Altman plot for diastolic blood pressure (DBP). The plot shows the agreement between measurements from invasive radial arterial monitoring (DBP-INV) and the finger cuff (DBP-FC). Participants’ multiple measurements are presented individually as participant number. The middle horizontal line indicates the bias, the bottom and top lines are the 95% limits of agreement and shaded regions represent the 95% CIs for the lower and upper limits of agreement

Table 2 shows the values that were used to derive the modified Bland-Altman plots, which are shown in Fig. 2. There were two consistently and markedly outlying participants; 14 (INV measures higher than FC) and 21 (INV measures lower than FC). The mean differences (bias) indicated INV was higher than FC across all three blood pressure measurements, all also having large ranges of agreement. The bias was most marked for SBP, which was almost triple the pre-specified acceptable limit of ±5 mmHg. Although the DBP bias was within ±5 mmHg, the limits of agreement were wide (-18.6–23.8).

**Table 2:** Comparison of values for mean arterial pressure, systolic blood pressure and diastolic blood pressure obtained from the finger cuff compared with invasive arterial line. Summary of the calculated bias and 95% limits of agreement with 95% CIs calculated and used to create the modified Bland-Altman plots shown in Fig. 2, INV – FC

	Bias ± SD mmHg	Lower LOA (95% CI) mmHg	Upper LOA (95% CI) mmHg
Mean arterial pressure	5.2 ± 10.9	-16.0 (-22.0 to -11.9)	26.5 (22.3 to 32.4)
Systolic blood pressure	14.3 ± 14.1	-13.4 (-20.6 to -8.2)	42.0 (36.9 to 49.2)
Diastolic blood pressure	2.6 ± 10.8	-18.6 (-24.4 to -14.4)	23.8 (19.7 to 29.6)

*FC* finger cuff measurements, *INV* invasive measurements, *LOA* limits of agreement

**Fig. 3:**
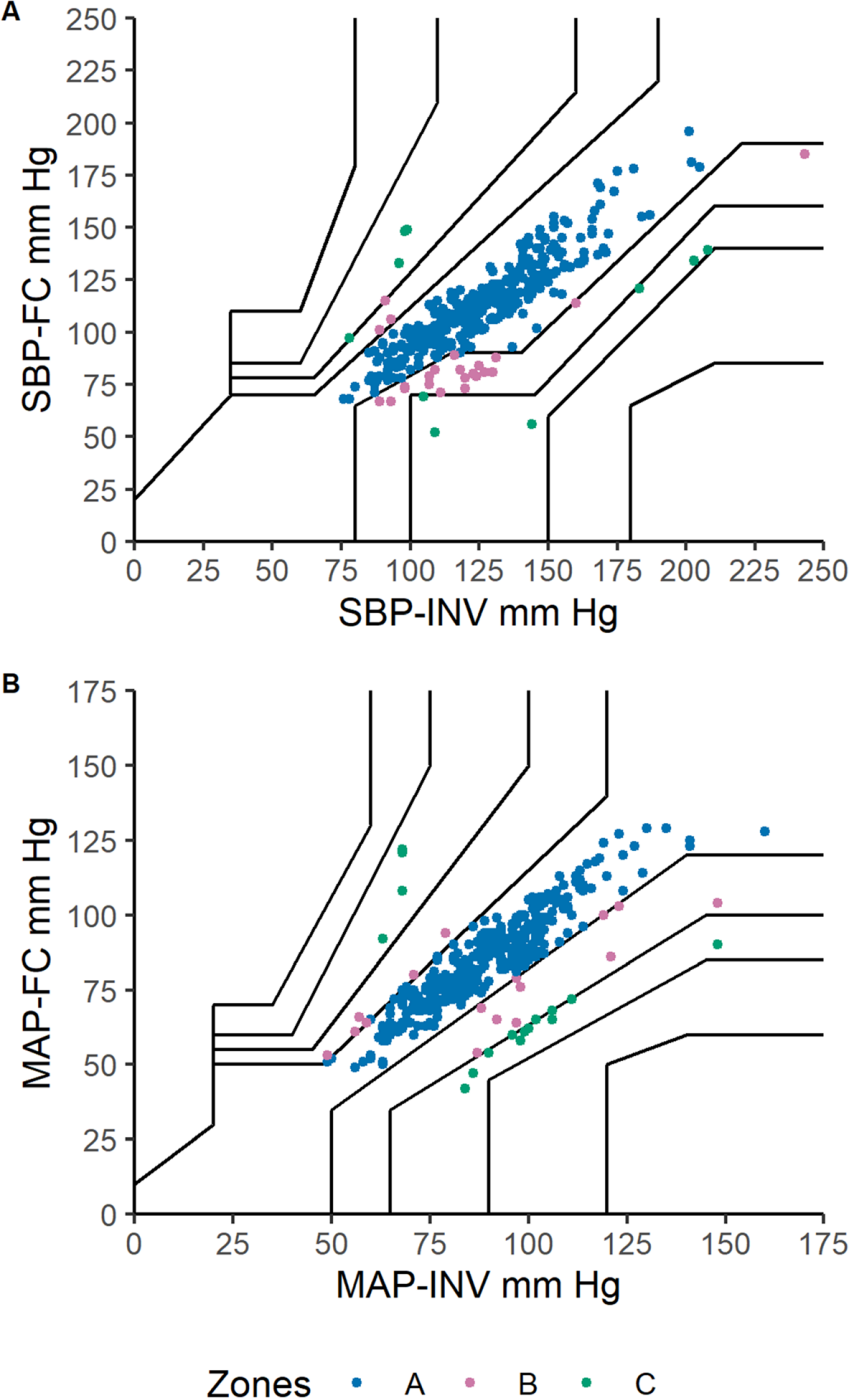
**a** Error grid analysis for systolic blood pressure (SBP). The figure shows the error grid for the test method (ClearSight™ finger cuff, FC) FC-SBP compared with the reference method (invasive radial arterial monitoring, INV) INV-SBP. **b** Error grid analysis for mean arterial pressure (MAP). The figure shows the error grid for the test method (ClearSight™ finger cuff, FC) FC-MAP compared with the reference method (invasive radial arterial monitoring, INV) INV-MAP

The results of the error grid analysis are shown in Fig. 3. For SBP, there were 336 (90.8%) of readings in Zone A, 24 (6.5%) in Zone B, 10 (2.7%) in Zone C and none in Zone D and E. For MAP, there were 338 (91.4%) in Zone A, 16 (4.3%) in Zone B, 16 (4.3%) in Zone C and none in Zones D and E.

**Fig. 4:**
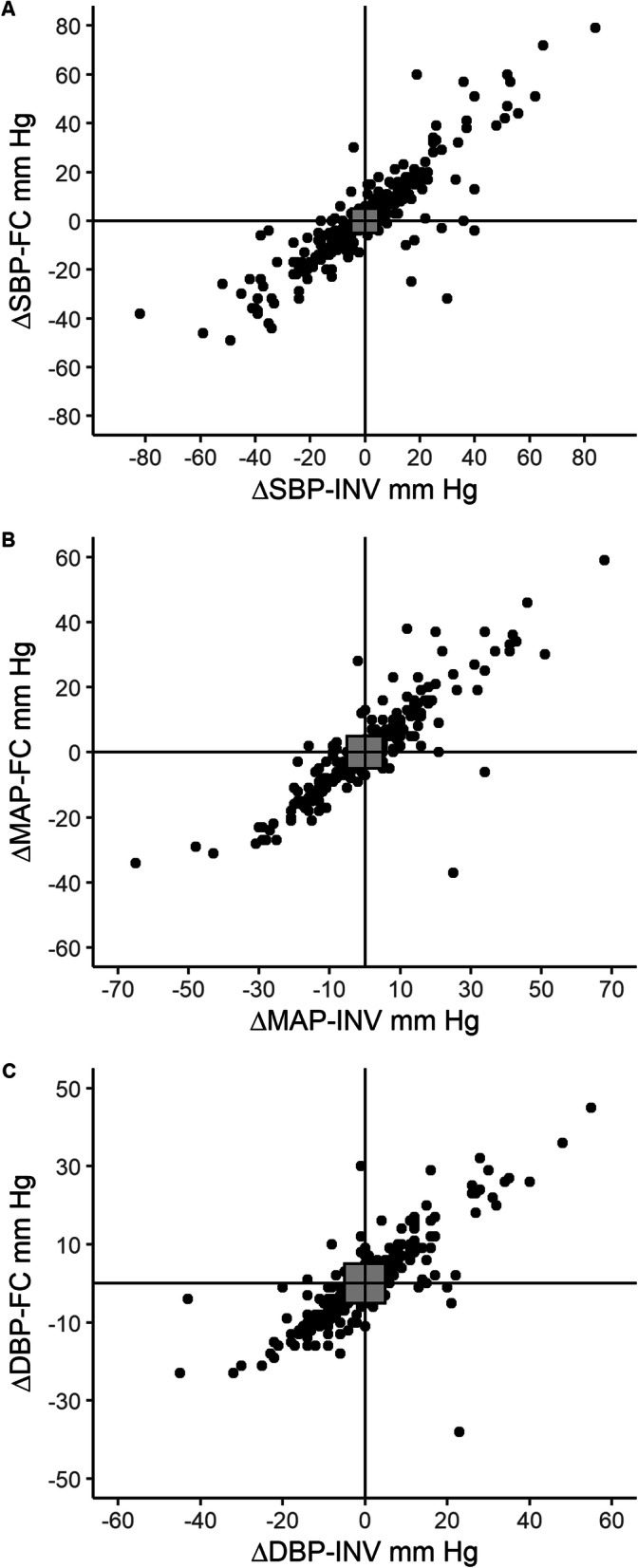
Four-quadrant plots of consecutive differences across time in **a** SBP, **b** MAP and **c** DBP, comparing measurements obtained from the ClearSight™ finger cuff (FC) and invasive radial arterial monitoring (INV). Grey squares indicate +/− 5 mmHg exclusion zone. Top left and bottom right quadrants indicate discordant pairs

The four-quadrant plots for SBP, MAP and DBP are shown in Fig. 4. There were 18 (7%) discordant pairs of 251 for SBP, 16 (8%) discordant pairs of 197 for DBP and 15 (7%) discordant pairs 215 for MAP.

## Discussion

When used intraoperatively in patients with BMI ≥45 kg/m^2^, the vascular unloading technique did not provide blood pressure measurements in agreement with those provided by the gold standard radial arterial monitoring. When measurements were assessed over time, we concluded that the FC could not be used interchangeably with radial arterial monitoring. The error grid analysis demonstrated that 97.3 and 95.7% of SBP and MAP readings were within the no- or low-risk zones (no or benign treatment). For MAP, the percentage of readings meeting the criteria for category C fell just outside that suggested as acceptable by Saugel et al. (4%) [19] and these represent a clinically meaningful number (26 of 740 readings). This would have led to moderate, non–life-threatening consequences in this high-risk population, all with a BMI ≥45 kg/m^2^. The proportion of discordant pairs of readings for SBP, DBP and MAP were similar to those reported by Rogge et al. [10]. Based on our results, the use of the vascular unloading technique is not appropriate for use in patients with obesity when accuracy is required within ±5 mmHg. Under the conditions of general anesthesia, the FC provided lower blood pressure values for MAP, SBP and DBP when assessed over time and the bias was substantially smaller for MAP and DBP, than for SBP.

This pattern was also observed in the study by Rogge et al. [10]. In 35 patients (median, IQR BMI 47,42–53 kg/m^2^, Rogge et al. showed that the mean difference between these techniques was greater for the SBP, followed by the MAP and the DBP (bias 6.8, 1.1 and 0.8 mmHg respectively) [10]. In our cohort, (median, IQR BMI 50, 48.3–55.3 kg/m^2^) the bias was greater for all three (14.3, 5.2 and 2.6 respectively). The limits of agreement calculated from the Rogge data are consistent with ours. Schumann et al. evaluated the finger cuff in 90 patients with a mean (SD) BMI of 48 (7) kg/m^2^. Their results, in patients with a mean (SD) mid-arm circumference of 43 (5) cm were more favourable towards the finger cuff [11]. Our results differed to those reported by both Rogge et al. [10] and Schumann et al. [11], in which the error grid analysis suggested minimal clinical risk resulting from use of the finger-cuff. Error-grid analysis is an emerging methodology in blood pressure measurement studies and is not yet a component of usual validation protocols [17]. However the provision of clinical risk stratification by the analysis is particularly relevant to anaesthetists, who make rapid, patient-specific decisions regarding blood pressure interventions during surgery.

Pre-operatively, the detection of hypertension in patients with obesity warrants investigation and optimisation [20]. In this context, the difference of ±5 mmHg in a blood pressure measurement is significant, determining the prescription (or not) of antihypertensive medication [20]. Equally important in post-operative period is the detection of hypotension, which may indicate haemorrhage, sepsis or cardiac dysfunction. Intraoperative hypotension and to a lesser extent, hypertension, have been associated with poor perioperative outcomes [21]. Accurate intraoperative measurements are necessary to ensure safe and appropriate interventions by the anaesthetist.

Based on their arm morphology, our cohort is representative of patients likely to have erroneous NIBP readings and for whom an accurate alternative is required [1, 4]. Three participants had a mid-arm circumference outside the oscillometric NIBP cuff range provided by our institution [22]. The shape of our participants’ arms was also extreme, with a left arm mean conicity index of 15.3, range 8.5 to 22.7. In comparison, a population of 450 pregnant women (representing the full range of BMI) had a much lower mean conicity index of 6.4 with a range of − 1.4 to 15.3 [23]. While offering the potential advantage of NIBP measurement that negates the extremes of arm morphology, use of the FC in this clinical context risks failure to detect significant systolic hypertension and thus may compromise clinical care. Another significant limitation is the failure of the ClearSight device to fit the large fingers of four participants in our cohort, an issue that has been previously identified [24].

Pouwel and colleagues failed to validate Nexfin™ in patients with obesity when compared to auscultatory sphygmomanometry [25]. A limitation of their study was failure to report the range of mid-arm circumference and the range of cuff sizes used. This limitation was also observed in Schumann et al. [11], where the applied NIBP cuff was a standard large size, not based on the mid-arm circumference, as recommended by the American Heart Association [26]. In 100 obese patients with mean (SD) mid-arm circumference of 42 (6.1) cm (similar to Schumann et al. [11]), we demonstrated that 11 would require an adult cuff, 61 would require a large cuff and 21 would require an extra-large cuff, with 7 having a mid-arm circumference larger than the recommended range according to the American Heart Association [4]. It is likely that many participants in the study by Schumann et al. should have been allocated a different cuff and this compromises their results [11]. Validation according to arm size will be a requirement of the proposed single universal standard for device validation [2]. Validation protocols must accommodate new devices and appropriate devices are required for use in patients with obesity [27, 28].

Our study adds to the currently limited understanding of the clinical performance of the vascular unloading technique, compared with invasive techniques, in patients who are very obese. Until recently, there were no studies evaluating the utility of this technique in this specific population. Our 30 participants were larger than the 35 evaluated by Rogge et al. [10] and larger than the 90 patients evaluated by Schumann et al. [11]. The mean mid-arm circumference of our cohort also significantly larger than that described in the cohort of Schumann et al. [11]. Our study has the added benefit of describing the arm morphology of our participants, identifying them as individuals in which NIBP measurement is notoriously difficult [1].

Our study has limitations. Female participants were over-represented in our group, reflecting our national bariatric registry sex differential and this has been previously noted locally [1]. Our results may have been compromised by use of the large finger cuff in four participants in whom the finger circumference was larger than that recommended by the manufacturer. Digital arterial flow (and thus the FC readings) may have been influenced by cannulation of the radial artery or by the administration of vasopressor infusion, which were used in most participants. The single-site nature of our study limits the generalisability of the results. Two participants could be identified on the modified Bland-Altman plots as being far outside the limits of agreement across for MAP, SDP and DBP. We could not identify a reason for these extreme readings when the BMI and vasopressor requirements of the two individuals were reviewed. Comparison of the FC with automated oscillotonometric NIBP cuff readings would have added to our study. Our study was undertaken in supine anaesthetised patients and the results are not necessarily generalisable to awake patients. As a pilot study, our analysis is limited by the small sample which introduces the potential for bias. Further evaluations in larger numbers of patients with BMI ≥45 kg/m^2^ are required.

This study also raises known difficulties in validating non-invasive continuous blood pressure monitoring devices, particularly in patients with obesity [28]. Current guidelines do not provide protocols for the validation of continuous NIBP devices [2]. The shape of the arterial waveform changes as it travels to the periphery, due to reflections at the arteriolar level [29]. Invasive arterial monitoring obtains the pressure at the radial artery [30], while the FC reconstructs the brachial waveform from pressure measured at the finger, using a proprietary algorithm [30, 31]. This is consistent with our findings that the difference in SBP between the two techniques was most affected, with SBP increasing as the pressure wave moves distally. Despite being applied in other studies [7–9] and there being no reasonable alternative, the limitations of radial arterial catheterisation as the gold standard for device validation should be considered. Validation against invasive monitoring is restricted to anaesthetised participants, those undergoing coronary catheterisation or receiving critical care [28].

## Conclusion

An increasing body of work is emerging regarding alternatives to oscillometric NIBP cuff measurements of blood pressure in patients with BMI ≥45 kg/m^2^. In this pilot study, the vascular unloading technique did not provide accurate blood pressure measurements when assessed over time, with the FC tending to provide lower values. The clinical consequences of these errors would have led to inappropriate interventions of moderate risk in a small but arguably significant fraction of readings, in this population with a high burden of comorbidities. Equipment suitable for patients with obesity is required for all perioperative phases and when invasive monitoring is not appropriate. Further evaluation of this device is required in larger numbers of patients with obesity.

## Data Availability

The dataset generated during and analysed during the current study are not publicly available due to them containing potentially identifying information but are available from the corresponding author on reasonable request”.

## Abbreviations

AAMI: Association for the Advancement of Medical Instrumentation
DBP: Diastolic blood pressure
FC: Finger cuff
INV: Invasive radial arterial blood pressure monitoring
MAP: Mean arterial pressure
MOVER: Method of variance estimates recovery
NIBP: Non-invasive blood pressure
SBP: Systolic blood pressure
