# Correction: ClearSight™ finger cuff versus invasive arterial pressure measurement in patients with body mass index above 45 kg/m^2^

**DOI:** 10.1186/s12871-023-02034-y

**Published:** 2023-03-10

**Authors:** Victoria Eley, Rebecca Christensen, Louis Guy, Kerstin Wyssusek, Anita Pelecanos, Benjamin Dodd, Michael Stowasser, Andre van Zundert

**Affiliations:** 1 Department of Anaesthesia and Perioperative Medicine, The Royal Brisbane and Women’s Hospital, Butterfield St, Herston, Queensland 4006 Australia; 2 Faculty of Medicine, The University of Queensland, St Lucia, Queensland 4067 Australia; 3 Statistics Unit, Queensland Institute of Medical Research Berghofer, Herston, Brisbane 4006 Australia; 4 Department of Surgery, The Royal Brisbane and Women’s Hospital, Butterfield St, Herston, Queensland 4006 Australia; 5 Hypertension Unit, Princess Alexandra Hospital, Woolloongabba, Brisbane 4102 Australia

**Correction: BMC Anesthesiol 21, 152 (2021)**

**https://doi.org/10.1186/s12871-021-01374-x**

Following publication of the original article [1], we provide here additional analyses and results pertaining to non-invasive blood pressure measurements recorded during the study. Upper arm non-invasive blood pressure (NIBP) measurements from 29 of the 30 participants were recorded during the study. It was subsequently identified that the arm cuffs used did not comply with the manufacturer’s recommendations for using a particular cuff size for the specific patient arm circumference [2]. Therefore comparative analyses were not included in the original reporting of this methods comparison analysis. For this reason we urge caution in drawing any conclusions from interpretation of these results.

The methods for obtaining invasive blood pressure measurements from the radial artery (INV) and non-invasive measurements from the ClearSight™ finger cuff (FC) have been published previously [1]. According to institutional protocol, the NIBP cuff was allocated by pre-operative nursing staff and approved by the anesthesia team. The institution uses Welch Allyn Flexiport™ Reusable cuffs (Welch Allyn Australia P/L, Macquarie Park NSW, Australia) which come in size 11 (adult: to fit a mid-arm circumference 25 – 24 cm), size 12 (large adult: 32 – 43 cm) and size 13 (thigh: 40 – 55 cm). There is no cuff size available for a mid-arm circumference >55 cm [2]. The NIBP cuff was placed on the upper arm on the same side as the radial artery catheter, except for cases in which this was not possible due to placement of intravenous access. NIBP measurements were obtained using an E-PSMP Carescape Module™ (GE Healthcare, Chicago, IL, USA).

Systolic blood pressure (SBP), diastolic blood pressure (DBP) and mean arterial pressure (MAP) were recorded digitally at 5-minute intervals from the initial blood pressure measurement for each patient for up to one hour, or until cessation of anesthesia, whichever occurred first.

Data from 29 participants were analysed. The correct cuff was allocated in two (7%) patients, according to the manufacturer’s recommendations and based on the measured mid-arm circumference. Of the incorrectly allocated cuffs, all were too small. In two participants, the arm circumference was outside the range provided for by the manufacturer. Eight (28%) patients had ipsilateral NIBP and INV arm measurements. Patients had complete data until 35 minutes of general anesthesia. Thereafter it ranged between 24 and 28 readings per method, as the length of surgery differed between patients.

We firstly present here the output of the analysis comparing the measurements obtained from the ClearSight™ finger cuff and the NIBP upper arm cuffs. Table 1 shows the results of the modified Bland-Altman analysis comparing these two methods. Figure 1 shows the Bland-Altman plots of MAP, SBP and diastolic blood pressure DBP comparing these two methods. Figure 2 shows the four-quadrant plots for SBP, MAP and DBP. An error-grid analysis was not undertaken, as this analysis requires one of the methods to be a gold-standard technique.

**Table 1:** Comparison of values for mean arterial pressure, systolic blood pressure and diastolic blood pressure obtained from non-invasive measurements arm cuffs (NIBP) compared with the finger cuff (FC) measurements in 29 participants (356 pairs of NIBP-FC measurements). Summary of the calculated bias and 95% limits of agreement with 95% confidence intervals calculated and used to create the modified Bland-Altman plots shown in Additional Figure 1, NIBP – FC

NIBP-FC	Bias (SD) mmHg	Lower LOA (95% CI) mmHg	Upper LOA (95% CI) mmHg
SBP	14.9 (17.5)	-19.3 (-29.4 – -12.3)	49.2 (42.2 – 59.3)
DBP	4.3 (11.5)	-18.3 (-24.4 – -14.1)	26.9 (22.6 – 33.0)
MAP	8.9 (11.8)	-14.3 (-20.7 – -9.8)	32.1 (27.6 – 38.5)

*INV* invasive measurements, *NIBP* non-invasive blood pressure measurements, *LOA* limits of agreement

**Fig. 1:**
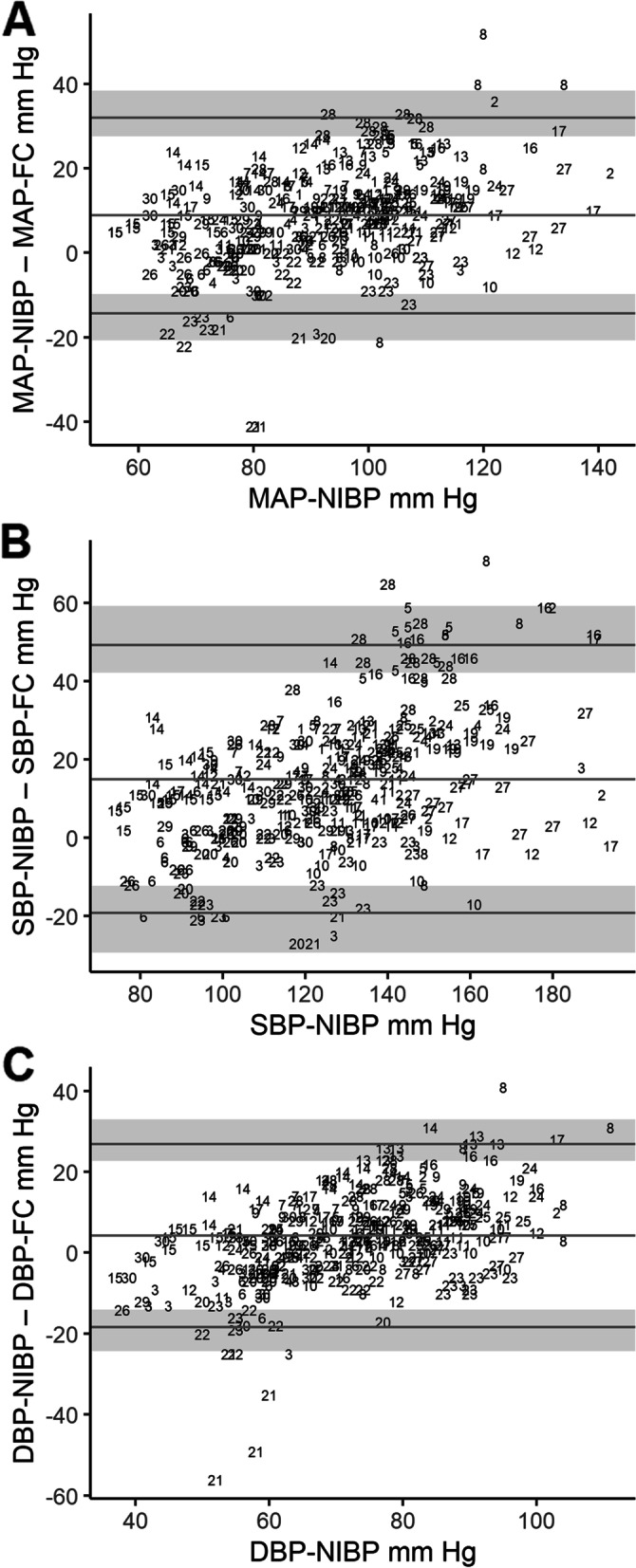
**A**. Modified Bland-Altman plot for mean arterial pressure (MAP). The plot shows the agreement between measurements from non-invasive arm cuffs (MAP-NIBP) and the finger cuff (MAP-FC). **B**. Modified Bland-Altman plot for systolic blood pressure (SBP). The plot shows the agreement between measurements from non-invasive arm cuffs (SBP-NIBP) and the finger cuff (SBP-FC). **C**. Modified Bland-Altman plot for diastolic blood pressure (DBP). The plot shows the agreement between measurements from non-invasive arm cuffs (DBP-NIBP) and the finger cuff (DBP-FC). Participants’ multiple measurements are presented individually as participant number. The middle horizontal line indicates the bias, the bottom and top lines are the 95% limits of agreement and shaded regions represent the 95% confidence intervals for the lower and upper limits of agreement

**Fig. 2:**
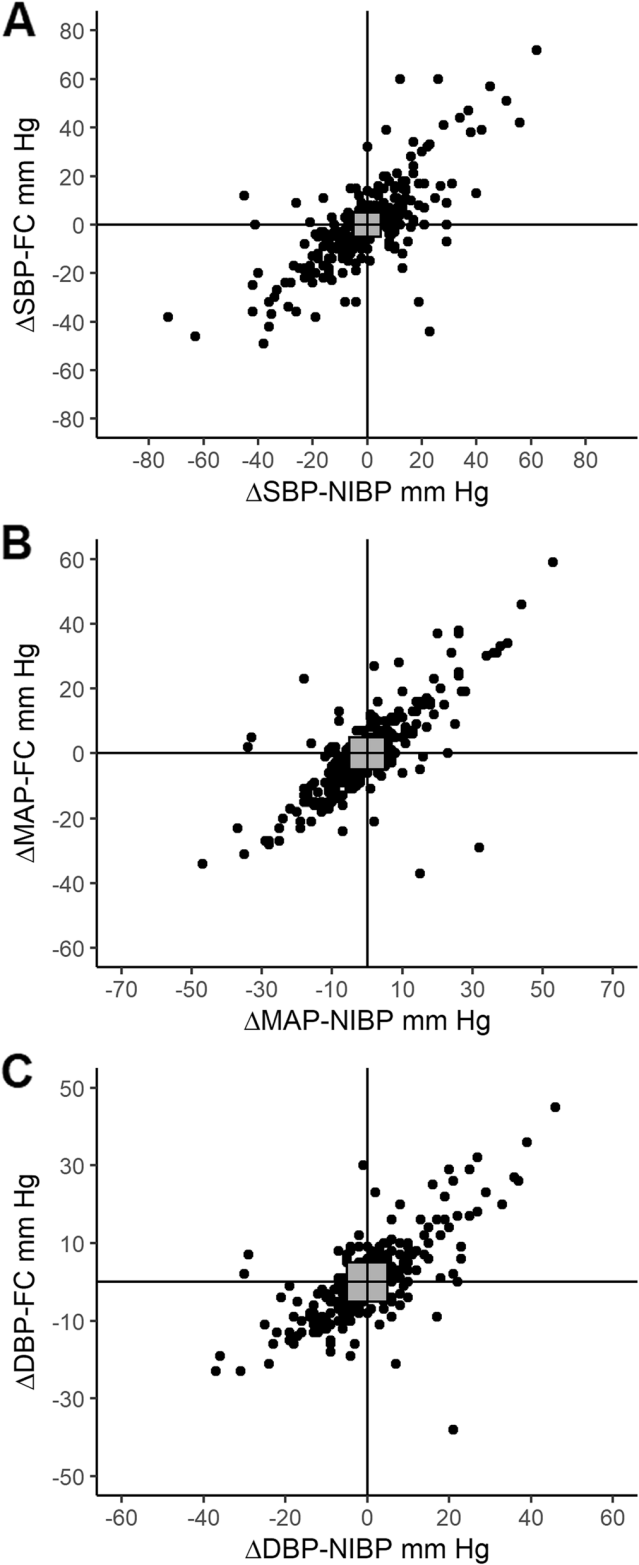
Four-quadrant plots of consecutive differences across time in **A**. systolic blood pressure (SBP), **B**. mean arterial pressure (MAP) and **C**. diastolic blood pressure (DBP), comparing measurements obtained from the ClearSight™ finger cuff (FC) and non-invasive arm cuffs (NIBP). Grey squares indicate +/− 5 mmHg exclusion zone. Top left and bottom right quadrants indicate discordant pairs

We also present the output of the analysis comparing the measurements obtained from the invasive arterial monitoring with the measurements obtained from the NIBP arm cuffs. Table 2 shows the modified Bland-Altman analysis comparing these two methods. Figure 3 shows the Bland-Altman plots of MAP, SBP and DBP comparing these two methods. Figure 4 shows the four-quadrant plots for SBP, MAP and DBP. Figure 5 shows the error grid analysis comparing SBP and MAP measurements from INV and NIBP.

**Table 2:** Comparison of values for mean arterial pressure, systolic blood pressure and diastolic blood pressure obtained from the non-invasive measurements arm cuffs (NIBP) compared with invasive arterial line (INV) in 29 participants (359 pairs of NIBP-INV measurements). Summary of the calculated bias and 95% limits of agreement with 95% CIs calculated and used to create the modified Bland-Altman plots shown in Additional Figure 3, INV – NIBP

INV-NIBP	Bias (SD) mmHg	Lower LOA (95% CI) mmHg	Upper LOA (95% CI) mmHg
Systolic blood pressure	-0.6 (16.8)	-33.5 (-43.8 – -26.4)	32.4 (25.2 – 42.7)
Diastolic blood pressure	-1.7 (10.6)	-22.5 (-28.5 – -18.2)	19.1 (14.9 – 25.2)
Mean arterial pressure	-3.6 (10.8)	-24.9 (-30.8 – -20.7)	17.6 (13.5 – 23.5)

*INV* invasive measurements, *NIBP* non-invasive blood pressure measurements, *LOA* limits of agreement

**Fig. 3:**
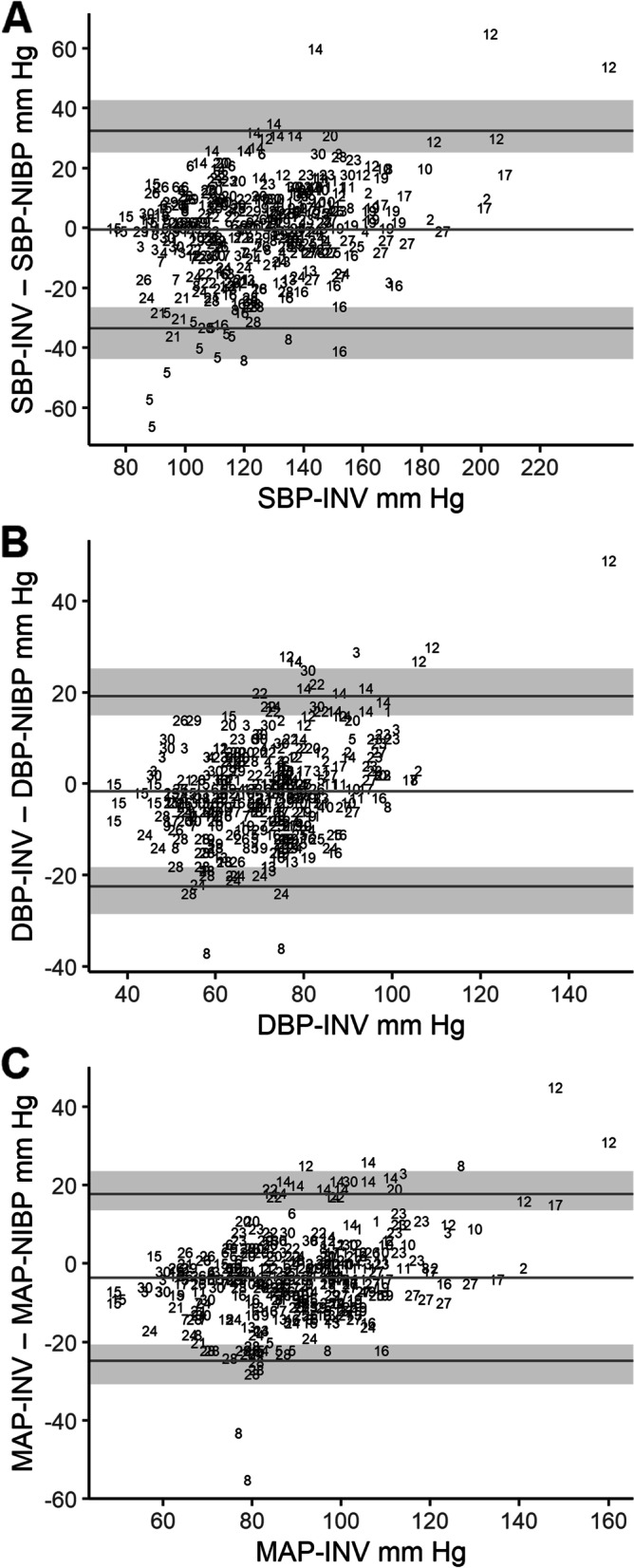
**A**. Modified Bland-Altman plot for systolic blood pressure (SBP). The plot shows the agreement between measurements from invasive arterial monitoring (SBP-INV) and the non-invasive arm cuffs (SBP-NIBP). **B**. Modified Bland-Altman plot for diastolic blood pressure (DBP). The plot shows the agreement between measurements from invasive arterial monitoring (DBP-INV) and the non-invasive arm cuffs (DBP-NIBP). **C**. Modified Bland-Altman plot for mean arterial pressure (MAP). The plot shows the agreement between measurements from invasive arterial monitoring (MAP-INV) and the non-invasive arm cuffs (MAP-NIBP). Participants’ multiple measurements are presented individually as participant number. The middle horizontal line indicates the bias, the bottom and top lines are the 95% limits of agreement and shaded regions represent the 95% CIs for the lower and upper limits of agreement

**Fig. 4:**
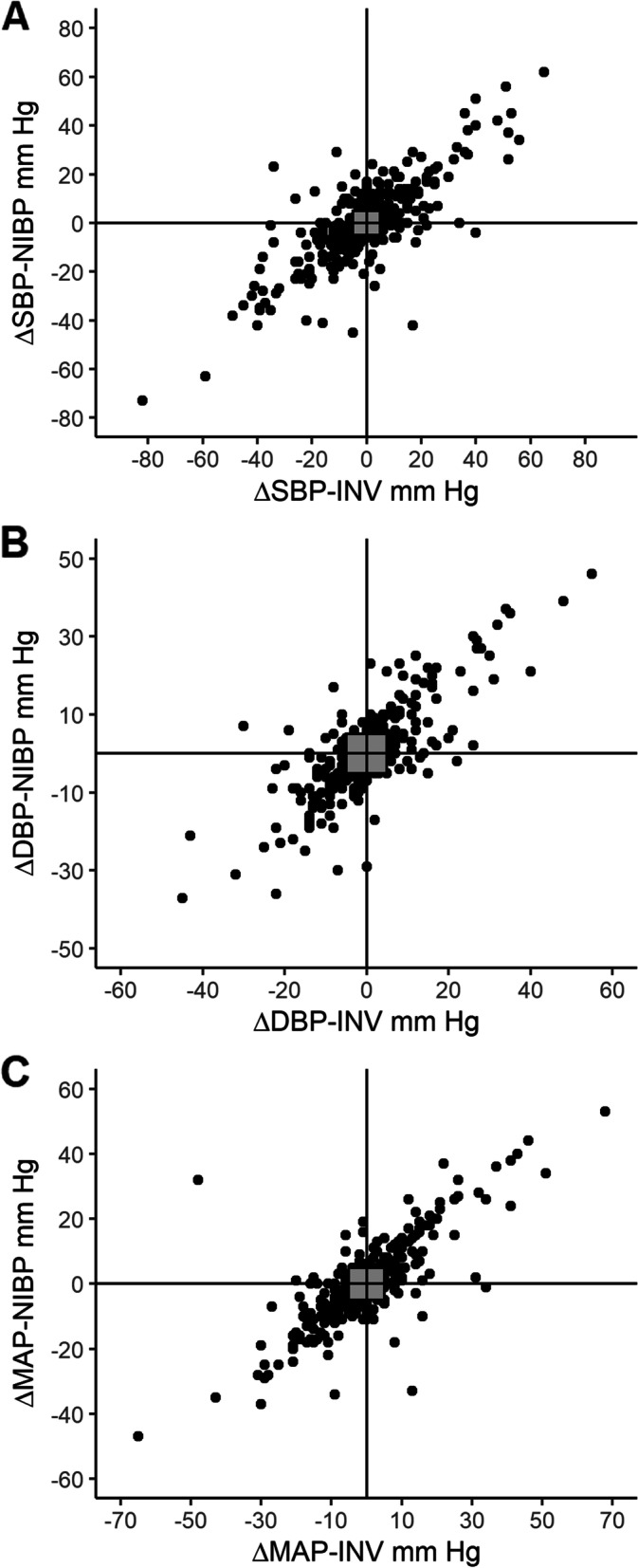
Four-quadrant plots of consecutive differences across time in **A**. systolic blood pressure (SBP), **B**. diastolic blood pressure (DBP) and **C**. mean arterial pressure (MAP), comparing measurements obtained from the non-invasive arm cuffs (NIBP) and invasive arterial monitoring (INV). Grey squares indicate +/− 5 mmHg exclusion zone. Top left and bottom right quadrants indicate discordant pairs

**Fig. 5:**
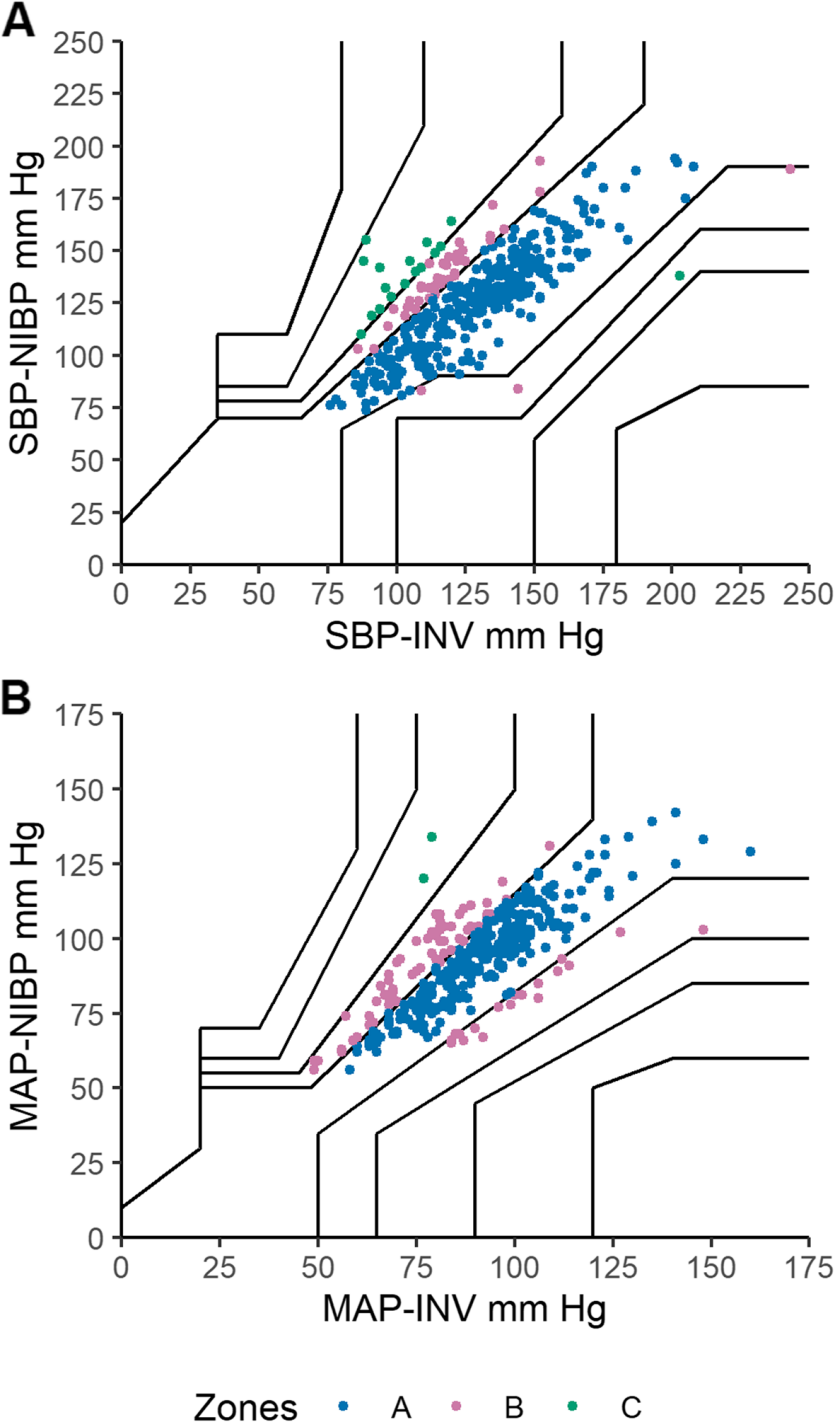
**A**. Error grid analysis for systolic blood pressure (SBP). The figure shows the error grid for the test method (non-invasive arm cuffs, NIBP) NIBP-SBP compared with the reference method (invasive radial arterial monitoring, INV) INV-SBP. **B**. Error grid analysis for mean arterial pressure (MAP). The figure shows the error grid for the test method (non-invasive arm cuffs, NIBP) NIBP-MAP compared with the reference method (invasive radial arterial monitoring, INV) INV-MAP

In a methods comparison analysis, each technique should be undertaken according to manufacturer’s recommendations. For this reason we urge caution in drawing any conclusions from interpretation of these results.

In the original paper we stated: “Comparison of the FC with automated oscillotonometric NIBP cuff readings would have added to our study.” This statement would have been better expressed “Comparison of the FC with accurately measured automated oscillotonometric NIBP cuff readings would have added to our study.” Our intention was to comply with recommendations of the European Society of Hypertension [3] and the Association of the Advancement for Medical Instrumentation [4], that were current at the time of our protocol design.
